# Compliance with eight years of annual ivermectin treatment of onchocerciasis in Cameroon and Nigeria

**DOI:** 10.1186/1756-3305-4-152

**Published:** 2011-07-27

**Authors:** William R Brieger, Joseph C Okeibunor, Adenike O Abiose, Samuel Wanji, Elizabeth Elhassan, Richard Ndyomugyenyi, Uche V Amazigo

**Affiliations:** 1 Department of International Health, The Johns Hopkins University Bloomberg School of Public Health, 615 N Wolfe Street, Baltimore, MD 21014 USA; 2 Department of Sociology/Anthropology, University of Nigeria, Nsukka, Enugu State, Nigeria; 3 Sightcare International, Ibadan, Oyo State, Nigeria; 4 Research Foundation in Tropical Diseases and Environment, Buea, Cameroon; 5 Sight Savers, Dakar, Senegal; 6 National Onchocerciasis Control Programme, Kampala, Uganda; 7 African Programme for Onchocerciasis Control, Ouagadougou, Burkina Faso

## Abstract

**Background:**

As the African Programme for Onchocerciasis Control (APOC) matured into its 10^th ^year of ensuring community involvement in mass annual treatment of onchocerciasis with ivermectin, there was recognition of a need to study not only annual coverage of ivermectin in villages but also the compliance of individual villagers with these annual treatments. This was based on the concern that while population coverage goals may be achieved each year, there might be segments of the population who systematically are not complying with the annual regimen, thus creating a reservoir of infection and threatening program gains.

**Methods:**

A multi-site study in five APOC sponsored projects in Nigeria and Cameroon was undertaken to identify the socio-demographic correlates of compliance with ivermectin treatment. A total of 8,480 villagers above 9 years of age selected through a systematic random sampling from 101 communities were surveyed to ascertain their levels of compliance, by adapting APOC's standard household ivermectin survey form. Community leaders, community directed distributors (CDDs) of ivermectin and health workers were interviewed with in-depth interview guides, while focus group discussions were held with community members to help explain how socio-demographic factors might affect compliance.

**Results:**

Eight-year compliance ranged from 0 to 8 times with 42.9% taking ivermectin between 6-8 times annually (high compliance). In bivariate analysis high compliance was positively associated with being male, over 24 years of age, having been married, not being Christian, having little or no formal education and being in the ethnic majority. These variables were also confirmed through regression analysis based on total times ivermectin was taken over the period. While these factors explained only 8% of the overall variation in compliance, ethnic status and education appeared to be the strongest factors. Those with higher education may be more mobile and harder to reach while neglect of ethnic minorities has also been documented in other programs.

**Conclusion:**

These findings can help managers of CDTI programmes to ensure ivermectin reaches all segments of the population equally.

## Background

The community directed treatment with ivermectin (CDTI) programme of the African Programme for Onchocerciasis Controls (APOC) was established in 1995. Its goal was to put in place a sustainable drug distribution system and maintain a minimum of 65% annual population coverage with Mectizan^® ^in endemic communities for at least 15 years, required for effective control of onchocerciasis [1-4]. Currently, CDTI is on-going in over 95,000 communities where over 98 million ivermectin tablets are distributed annually to treat over 33 million people [5]

While reports of population coverage are encouraging [5,6] coverage rates in a community may not give the full picture of program success because there may be individuals or groups who systematically do not comply over the years and thus, provide a continued focus for disease transmission. Compliance, also known as adherence, is defined as "the extent to which a patient acts in accordance with the prescribed interval and dose of and dosing regime [7]. Compliance with annual ivermectin treatment therefore, has become a major challenge for (APOC) as it enters its second decade of implementation on the ground. Considering that ivermectin treatment will be needed for many years into the future, the Technical Consultative Committee of APOC requested a study to learn what factors might be associated with compliance over time so that appropriate education and intervention could be designed to help sustain annual treatment [8].

To date, no published reports of CDTI have actually determined whether individual community residents actually comply with ivermectin treatment consistently each year. This may be due in part to the fact that in the early years of the program not enough annual distributions had accumulated to provide a meaningful measure of compliance. Now that the original 25 projects, which started in 1997-98, have been operating for almost a decade annual compliance studies have become possible. Such studies also are extremely desirable since researchers are now pushing back the timeframe for controlling onchocerciasis through annual ivermectin dosing from 15 to 25 or more years [9]. APOC has a mandate to establish within a period of 12 to 15 years, effective and sustainable, community-directed treatment with ivermectin throughout the endemic areas within the geographic scope of the programme (APOC, 2006), and thus requires a clear understanding of the long-term compliance process in order to guide countries toward sustainability.

There are studies about the factors that encourage or discourage taking ivermectin during specific distributions such as age, gender and ethnicity [10-16]. Social support and drug perceptions are other factors that have influenced coverage, and hence may impact on compliance [17]. Akogun *et al*. (2000) discussed the importance of perceived benefits of ivermectin treatment, which could be another motivating factor in annual compliance [18]. These factors may or may not explain long term compliance. APOC therefore wanted a scientific basis to frame health education and communication that will promote continued compliance with CDTI.

Compliance studies on other medical conditions have shown that adhering to a medical regimen may be influenced by the characteristics of the patient/client and provider as well as the nature of the regimen and may guide thinking about compliance with ivermectin treatment. Considering the question of whether specific segments of the population may be systematically not complying, one notes that ethnic origin and educational level have been associated with compliance [19,20].

The study of compliance rates was explored in five CDTI projects in two countries, Nigeria and Cameroon, where annual ivermectin distribution had happened at least eight times. This paper documents the compliance rate among villagers who were old enough to begin ivermectin treatment at the start of these projects and some of the possible socio-demographic characteristics associated with compliance levels. Results are expected to contribute to developing a simple and efficient protocol for determining compliance rate at regular intervals, which could be used by National Onchocerciasis Taskforces for periodic monitoring of compliance to treatment with ivermectin in their respective project areas.

## Methods

### Study Design and Area

The study was designed to determine rates of compliance to and perceived benefits of annual ivermectin treatment for the control of onchocerciasis in five APOC supported project sites in Cameroon and Nigeria. The cross-sectional approach was adopted in collecting quantitative and qualitative data in study sites, which had been screened through the feasibility study conducted in 2005 that verified the availability of village based adequate records to document eight continuous years of ivermectin distribution. The five sites included Kaduna, Cross River, Imo and Taraba States in Nigeria and the Southwest Province in Cameroon, places where CDTI projects have been providing ivermectin annually since 1998.

### Study Population and Sampling

The study population resided in villages where ivermectin distribution has occurred using the CDTI approach for at least eight consecutive years. The survey focused on household members in these villages who were old enough to have started receiving ivermectin at the beginning of the program. At each site researchers balloted for 2 districts among those that were found to have adequate opportunity for longer-term compliance resulting in a random sample of 10 villages per district.

The first step on reaching a village was obtaining and reviewing the village distribution records. Village records yielded data on whether distribution took place, the level of coverage each year and the number of times individual household members took ivermectin. Records were also studied to determine whether there had been continuous annual ivermectin distribution for eight years. If this was not the case, another village was sampled as a substitute.

Households ranged from 5-6 people in the rural areas covered. A goal of 300 households was chosen per study site. Village registers/records maintained by the CDDs were used to identify and ballot for 15 households in each village. All resident household members included in the household survey form.

The sample population for the qualitative component consisted of community leaders, Community Directed Distributors (CDDs: volunteers chosen by their communities), and health workers for in-depth interviews, female and male adults, youth and ethnic minority community members who were included in focus group discussions.

### Data Collection and Management

The household survey form was adapted from the standard household coverage forms used in CDTI program assessments by APOC. All selected households were visited and the purpose of the survey was explained to all members present. Their permission was obtained to continue with the survey. Those present were asked to name all currently resident household members and validate their age, sex, educational and ethnic characteristics. They were also asked about reasons any member did not take ivermectin during the most recent distribution and to estimate how many times each member had taken annual ivermectin treatment in all previous distributions. Once all information had been obtained in the household, the researchers returned to the CDD and filled in a column on the form for the number of times each household member was recorded as having taken ivermectin in the village register. Treatment data from the registers was used for analysis of compliance rates to avoid recall bias.

Qualitative instruments consisted of in-depth interview guides for village leaders, CDDs and health staff at the nearest health facility. Twenty focus group discussions were held in each site. These were divided among male and female adults, youth and ethnic minorities and between the two sample districts. The population used for FGDs was alternated among the selected villages such that only one FGD was held in each village

One hundred focus group discussions (FGDs) were held. In-depth interviews took palce with 150 community leaders, 100 CDDs as well as 10 health workers at each site. Finally, field notes compiled by research staff on a daily basis also provided the context of the information gathered for the study.

All quantitative data were processed and analyzed with the EPI INFO version 6d. Standard data entry templates were prepared for all quantitative data collection instruments. Simple descriptive statistics were employed in characterizing the respondents while some socio-demographic and key perceptual factors were employed in regression models constructed to explain levels of compliance among the respondents.

All qualitative data transcripts were typed using standard word processing software. A computer-assisted analysis of these data was undertaken using ATLAS.ti. Illustrative quotes were extracted from coded domains to explain the results of the quantitative analysis. Researchers from all sites participated in a final data analysis workshop.

### Ethical Considerations

Ethical clearance was obtained from ethical clearance committees of the University Teaching Hospitals in Enugu, Kaduna in Nigeria and Buea in Cameroon. Permissions were also sought from the community leaders. An informed verbal consent was obtained from every eligible individual before inclusion into the study by explaining the objective of the research. Privacy and confidentiality were ensured.

After the interview, data collectors provided important information regarding onchocerciasis mainly focusing on misperceptions and knowledge deficits observed during the interview with the respondents.

## Results

Coverage data were obtained from the 101 selected villages to ascertain that distribution had taken place. A brief look at the three years prior to the study is instructive. Mean population coverage rates were 70% in 2003, 70% in 2004 and 74% in 2005. The proportion of villages achieving the targeted 65% coverage in those years was 65%, 67% and 78% respectively.

### Overview of Household Survey

Among the 10,088 persons recorded on the household survey forms, 23.9% came from Cameroon, and 76.1% from Nigeria. Those above 9 years of age comprised 8,606 or 85.3% of the total, and this number became the total used in further analysis. Among those above 9 years of age the portion of females was 50.7%. The sample was nearly equally divided between currently and ever-married people (43.1%) and single ones (56.9%). The majority (95.4%) reported observance of the Christian faith, 3.0% were Muslim, while 1.6% claimed indigenous African and other religious practices. The smallest proportion of respondents (13.9%) had no formal education, most had either primary (53.1%) or post-primary (33.0%) education. Finally, 5.0% of this sample was ethnic minorities.

Of this sample 80.7% had swallowed ivermectin tablets during the last treatment round. Most (94.0%) could recall a number of times ever taken, which ranged from 0 to 8 with an average of 4.6. The number of times treatment was received according to CDD ranged from 0 to 8 with a mean of 4.1.

Only 67.2% of people recalled the same number of treatments received as that recorded in the register. Some (16.2%) thought they had taken ivermectin more times, while another 16.2% recalled fewer treatments than were recorded. This difference between recall and records, was also found in the feasibility study, and prompted the researchers to use CDD records as the standard for calculating compliance.

Nearly 5,700 people mentioned reasons for not taking tablets at some point in the past. These included the usual exclusion criteria such as sickness (6.3%), pregnancy (8.8%), age (8.6%), and shortness (1.1%). Personal reasons included bring absent (57.9%), fear of side effects (1.8%), 'do not take western drugs' (0.1%), and refusal (20.5%). Programmatic problems included not being informed (4.4%), the drug had finished (1.0%), and no distribution (9.2%).

**Figure 1:**
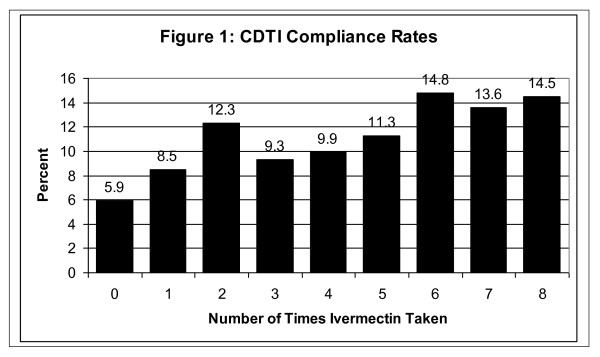
**CDTI Compliance Rates**.

Figure 1 shows the distribution of compliance over eight distributions. At the lower end, 26.7% had taken ivermectin only once, twice or not at all. A little less than one-third (30.5%) were in the middle range, having taken it 3-5 times. The largest number (42.9%) took ivermectin 6-8 times (defined as 'high compliance').

#### Compliance Analysis

Table 1 is a contingency table showing the comparison of socio-demographic factors with the three levels of compliance. Among females 41.1% were in the high compliance groups compared with 44.7% of males. Persons aged 25 years and older (adult) had a greater proportion of high compliers (47.4%) than the youth (37.1%). In a similar vein, 49.0% of those who had ever been married were high compliers compared with 36.8% of single people. Although Christians (95.4%) comprised the vast majority in the study area, only 41.8% were high compliers compared to 66.2% of those adhering to other religions.

**Table 1:** Demographic Factors and Compliance from Household Survey

Factor		Number	Level of Compliance (%)			X^2^, p value
				
			Low (0-2)	Moderate (3-5)	High (6-8)	
Sex	Male	4173	25.9	29.4	44.7	10.00
		
	Female	4296	27.4	31.4	41.1	0.004

Age	Youth (10-24 yrs)	3714	29.1	33.8	37.1	91.20
		
	Adult	4766	24.8	27.8	47.4	0.000000

Ever Married	Yes	4239	23.8	27.2	49.0	130.01
		
	No	4228	29.6	33.6	36.8	0.000000

Religion	Christian	8080	27.2	31.0	41.8	91.88
		
	Others	391	14.1	19.7	66.2	0.000000

Education	None	1395	17.8	22.4	59.7	208.36
		
	Primary	3506	26.6	31.7	41.7	0.000000
		
	Post-Primary	3565	30.1	32.3	37.6	

Ethnic	Majority	7984	24.5	31.1	44.4	380.14
		
	Minority	435	66.4	20.2	13.3	0.000000

Levels of high compliance decreased as educational level rose: 59.7% with no formal education had high compliance compared to 41.7% of those with at least primary education and 37.6% of those with secondary or higher education. Finally the proportion of high compliers among ethnic minorities in the study areas was much lower (13.3%) than that of the majority groups (44.4%).

Further analysis was done using compliance as a numerical value. Regression analysis confirmed the bivariate analysis as seen in Table 2. Males, adults and those who had ever been married had higher rates of compliance than females, youth and singles. Education level and Christianity were negatively associated with compliance, while belonging to the majority ethnic group in the area was a positive factor concerning compliance rate. Although educational level and ethnic status appear to be the strongest factors associated with compliance, overall these various socio-demographic variables explain only about eight percent of the variance in compliance rate.

**Table 2:** Analysis of Factors Associated with Compliance Rate

**Correlation coefficient:**		**r^2 = 0.08**	**ra^2 = 0.08**			
Source	df	Sum of Squares	Mean Square	F-statistic		

Regression	6	4078.0842	679.6807	119.17		
Residuals	8371	47743.9857	5.7035	.		

Total	8377	51822.0698				
B Coefficients						
		B	95% confidence			Partial
Variable	Mean	coefficient	Lower	Upper	Std Error	F-test

SEX	0.4930	0.2370708	0.132312	0.341829	0.053441	19.6795
AGE	30.9842	0.0105952	0.006414	0.014776	0.002133	24.6773
EVERMAR	0.5010	0.2147027	0.075348	0.354057	0.071089	9.1216
CHRISTIAN	0.9550	-1.1844426	-1.434186	-0.934699	0.127402	86.4327
EDUCATION	1.2613	-0.4355129	-0.512785	-0.358240	0.039419	122.0646
ETHNIC	0.9481	2.0099674	1.778477	2.241458	0.118090	289.7001

Y-Intercept		3.7916014				

#### Perspectives from Qualitative Interviews

Qualitative results were in large part normative and supported the idea that all eligible people in the community do take ivermectin regardless of their personal characteristics. In Nigeria male and female community leaders and CDDs agree that 'men, women and children take it'. Respondents explained that those who do not take it are pregnant women and children below the age of 5 years (i.e. only those not eligible). For example, in Taraba, Nigeria FGD participants said all eligible people take the drug; only those who are sick or below the age or height, as well as pregnant women are not given. In Cameroon CDDs also reported that those who take ivermectin are not sick, not pregnant and are above 5 years of age (all eligible).

There was some disagreement on sex differences. "I think men take it more regularly than women," said a FGD respondent in Cameroon). "Statistics shows that when distribution is done during the farming period, men take Mectizan^® ^more than women. Women are more afraid than men," was the observation of a Health Worker in Cameroon.

The age factor was also discussed. Some respondents support the idea that the elderly are more compliant. Cameroon FGD participants felt that old people like to take it more than younger because "they experience the problem of blindness." A female health worker in Imo State, Nigeria observed that mostly elderly people take it because "they are more in the village and know what the drug will do." In Taraba State, Nigeria a community leader reported that, "While everyone wants to take ivermectin, old people want it more."

A view from youth in Cameroon is that youth take it more because they are "better informed," and "it gives them strength for more work." A youth in Cross River State, Nigeria said, "They (youth) go for it more, as they have fewer side effects than the elderly."

In actuality, many people did not perceive age differences. Male FGD participants in Cross River and in Imo believe "everybody takes it if eligible, as it is good for everybody." In Kaduna a FGD participant thought that, "Anyone who was not pregnant or too short, take ivermectin, even the elderly."

Some occupational links were observed in the qualitative data. On the positive side, after taking the drug, "...farmers can go to their farms and work very well and produce crops" (Nigeria). A contrasting view was that, "Farmers may be weakened immediately they finish taking the drug because the drug makes you weak" (Cameroon). Disruption of work itself, not the drug was cited in another FGD where respondents noted that, "Others prefer to go for business or the farm than stay at home to collect the drug" (Nigeria).

While high compliance varies by ethnic status from the household survey, most discussants claimed there was no discrimination in providing ivermectin to minority groups. CDDs in Cameroon say they do not discriminate against 'strangers' living among them. A CDD in Kaduna noted Fulani living among them who take ivermectin because of health education. Another CDD in Kaduna confirmed that minority Fulani take it regularly because of health education. A third Kaduna CDD reported that minorities from other states take it because they see us doing so. In an FGD among minority Fulani in Kaduna participants said they receive ivermectin the same as the indigenous people.

Some minority issues did arise that could affect compliance. On a somewhat lighthearted note, a Kaduna CDD said, "Igbos also take it, but read the label first." A female FGD respondent in Cameroon said, "Everyone is welcome to it," but noted that, "Migrant farm laborers don't want it as may make them miss work." In Taraba, a Fulani woman explained that they take ivermectin only when it is brought to them (the program has not trained minority CDDs). A female health worker in Taraba observed that the main indigenous population "takes it more."

According to an FGD participant in Cross River 'travelers' do not want to take ivermectin as it may hamper their business. A Cross River CDD said minorities don't take it because they don't know when it is being distributed. A CDD in Cameroon said seasonal farmers only take it if the distribution period meets them while in the community. A Kaduna community leader explained that minorities do take the drug 'when they are around' One villager was not complimentary when saying, "Those who don't take the drug regularly are the Fulani because they are ignorant."

## Discussion

On average most of the villages have been obtaining population treatment targets of 65% and above in recent years, but that does not mean that individuals have been complying with annual treatment. Over one-quarter of age-eligible people in the community are low compliers and thus serve as a reservoir for continued transmission of onchocerciasis. The results of this study may help CDTI program managers target segments of the population with health education to increase compliance. Particularly, annual refresher training may be needed for CDDs to review with them compliance problems and encourage them to reach out to community members with low levels of compliance.

An important caveat should be considered, and this is the fact that these six socio-demographic factors explain a small proportion of the overall variance in compliance rates. Perceptual factors that may influence compliance are being explored in another component of this study.

In terms of coverage, other studies have shown a persistent difference between males and females. Even after controlling for the exclusion criteria of pregnancy, it has been found that women are less likely to take ivermectin at a given distribution [10,11]. What may happen is that if they are excluded once for pregnancy, they may be less likely to take ivermectin again the next year [11]. Ethnic minority status is relative. Another coverage survey found that minorities living in their own villages were more likely to take ivermectin during a distribution than minorities living within villages of the dominant ethnic group in the area [10].

Greater mobility may lie at the heart of three factors associated with low compliance. Younger people, those not yet married and those with higher education are likely to travel outside the village for work and other opportunities and miss annual distributions.

## Conclusion

Future research could test the effect of educational interventions on compliance now that a baseline of compliance exists as a result of this study. Another possible study would compare compliance with microfilaria levels. There has always been the assumption that annual treatment is the ideal, but in fact studies linking compliance and microfilaria levels might show that less frequent treatment may still be somewhat beneficial in terms of controlling transmission. Since CDTI will hopefully continue into the future, continued health education, program monitoring and operations research will be essential for sustaining high levels of both coverage and annual compliance.

## Competing interests

The authors declare that they have no competing interests.

## Authors' contributions

UVA conceived the study. All the authors participated in the design and execution of the study. WRB and JCO analyzed while WRB did the first draft of the paper. All contributed to the final paper.
